# Viral nanomotors for packaging of dsDNA and dsRNA

**DOI:** 10.1111/j.1365-2958.2007.05706.x

**Published:** 2007-05-01

**Authors:** Peixuan Guo, Tae Jin Lee

**Affiliations:** Department of Comparative Pathobiology and Weldon School of Biomedical Engineering, Purdue University West Lafayette, IN 47907, USA

## Abstract

While capsid proteins are assembled around single-stranded genomic DNA or RNA in rod-shaped viruses, the lengthy double-stranded genome of other viruses is packaged forcefully within a preformed protein shell. This entropically unfavourable DNA or RNA packaging is accomplished by an ATP-driven viral nanomotor, which is mainly composed of two components, the oligomerized channel and the packaging enzymes. This intriguing DNA or RNA packaging process has provoked interest among virologists, bacteriologists, biochemists, biophysicists, chemists, structural biologists and computational scientists alike, especially those interested in nanotechnology, nanomedicine, AAA+ family proteins, energy conversion, cell membrane transport, DNA or RNA replication and antiviral therapy. This review mainly focuses on the motors of double-stranded DNA viruses, but double-stranded RNA viral motors are also discussed due to interesting similarities. The novel and ingenious configuration of these nanomotors has inspired the development of biomimetics for nanodevices. Advances in structural and functional studies have increased our understanding of the molecular basis of biological movement to the point where we can begin thinking about possible applications of the viral DNA packaging motor in nanotechnology and medical applications.

## Introduction

Viral genomes are enclosed in a protein shell called capsid. After synthesis by separate machinery, viral structural proteins and the genome must interact with each other to form a complete virion through a process referred to as DNA or RNA packaging. For rod-shaped single-stranded DNA viruses (e.g. phage M13, F1) or single-stranded RNA (ssRNA) viruses (e.g. tobacco mosaic virus), the capsid proteins are usually assembled around the viral genome. Thus, the size of the virus is proportional to its genome size. However, all linear double-stranded (ds)DNA or dsRNA viruses, including dsDNA bacteriophages (Black, 1989; Guo, 1994), adenoviruses (Zhang and Imperiale, 2000), poxviruses (Koonin *et al*., 1993), human cytomegaloviruses (HCMV) (Scheffczik *et al*., 2002), herpes simplex viruses (HSV) (Salmon and Baines, 1998) and dsRNA bacteriophages (Olkkonen *et al*., 1990), possess a common feature in that their genome is packaged into a preformed procapsid. This entropically unfavourable process is accomplished by an ATP-driven packaging motor (Earnshaw and Casjens, 1980).

One objective in viral packaging motor studies is to understand the mechanical and physical behaviour of the motor complex and individual components. Attempts at direct or indirect observation of the physical behaviour of these motors aim to provide answers to questions concerning motor mechanisms, including (i) which component binds ATP to convert chemical energy into physical motion; (ii) how the motion starts and continues; (iii) how different parts in the motor respond to applied force and (iv) how conformational changes of each component are related to the force generation. Some of the answers pertaining to the above questions have been addressed in two comprehensive reviews more than 16 years ago (Earnshaw and Casjens, 1980; Black, 1989). This review focuses on the structure and function of motors of dsDNA viruses, while the recent exciting findings in genome packaging of dsRNA viruses are also introduced. It will also discuss how advances in the study of viral DNA packaging motors have pioneered or will facilitate the application in nanotechnology and medicine. The relationship between viral DNA/RNA packaging motors and other systems involving DNA replication, RNA transcription, macromolecule interaction and cross-membrane transportation is also discussed.

## Essential components of viral DNA packaging motor

Although other structural components of viral procapsids have been reported to be involved in DNA translocation, the essential components of the motors include a channel and DNA packaging enzymes. In tailed bacteriophages, the channel is generated via the assembly of a DNA-translocating oligomeric ring portal, called the connector, embedded in the pentagonal portal vertex of the procapsid (Bazinet and King, 1985; Jimenez *et al*., 1986; Guasch *et al*., 2002). The non-structural DNA packaging enzymes are also called ‘terminases’ for those viruses that replicate their genome into concatemeric DNA. The terminase possesses the capacity to cut concatemeric DNA after packaging. In bacteriophage phi29, one of the DNA packaging enzymes is a pRNA (packaging RNA) (Guo *et al*., 1987a).

### DNA translocating portal or the connector

**Fig. 1:**
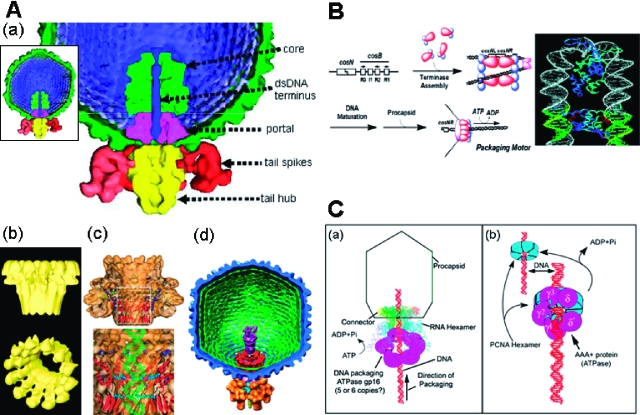
Viral DNA packaging motors. A. The portal or the connector structure of bacteriophage ε15 (a) (Jiang *et al*., 2006), T3 (b) (Valpuesta *et al*., 2000), T7 (c) (Agirrezabala *et al*., 2005) and P22 (d) (Lander *et al*., 2006). B. The assembly pathway of λ terminase and the structure of the related DNA substrate (Maluf *et al*., 2006; Ortega and Catalano, 2006). C. Similarity between two models; the phi29 DNA packaging motor (a) and PCNA/clamp-loader complex (b) (Lee and Guo, 2006). Figures were adapted with permission from the authors and from Elsevier, American Association for the Advancement of Science, and American Chemical Society to the respective citation.

The bacteriophage connector is composed of 12 copies of single protein and forms a six- and 12-fold symmetric ring attached to a fivefold symmetric vertex of the outer shell that leads to a symmetry mismatch between the capsid and the portal. A similar dodecameric portal has also been found in HSV type 1 (Newcomb *et al*., 2001; Trus *et al*., 2004). As proposed (Hendrix, 1978), such a symmetric mismatch would enhance the free energy (ΔG) to facilitate the relative rotation of the two rings of the portal during DNA packaging, although it is still unclear whether the portal rotates during DNA translocation. The portal obviously plays an important role in packaging, as amino acid substitutions or N-terminal extensions on the portal protein dramatically block the packaging. Translocation of dsDNA molecules during maturation or infection occurs through a 3–4 nm channel that runs along the connector. The 3D structures of several isolated recombinant connectors have been determined by Cryo-EM (Jimenez *et al*., 1986; Lurz *et al*., 2001; Guasch *et al*., 2002; Fokine *et al*., 2004; Trus *et al*., 2004; Agirrezabala *et al*., 2005; Jiang *et al*., 2006; Lander *et al*., 2006) (Fig. 1A).

The structure of the phi29 connector exhibits a truncated cone-shaped, with a narrow end of 6.8 nm in diameter exposed to the outer solvent and a wider end of 13.8 nm that is buried within the capsid. The height of the connector is about 7.5 nm (Simpson *et al*., 2000; Guasch *et al*., 2002). Several lysine residues are exposed on the internal surface of the channel. The positive charges of their -NH2 groups probably play a key role in DNA translocation (Guasch *et al*., 2002). While portal proteins, the building blocks of the connector, from different viruses share little sequence homology and show large variations in molecular size, they possess a significant degree of morphological similarity (Fig. 1A). The structure of the T7 connector at 8 Å resolution reveals that an alpha-beta fold builds the channel (Agirrezabala *et al*., 2005), a common feature also found in the connectors of different viruses including phi29,T3,T4, SPP1, P22, ε15 and possibly HSV.

One crucial function of the connector is to serve as a nucleation point for the assembly of motor components. The binding of a large subunit of DNA packaging enzymes (see below) to the connector has been reported, including gpA in phage λ (Catalano *et al*., 1995), gp12 in phi21 (Feiss *et al*., 1985), gp17 in T4 (Rao and Black, 1988) and gp19 in T3 and T7 (Yamagishi *et al*., 1985; Morita *et al*., 1993), and both gp16 and pRNA of phi29 (Guo *et al*., 1987b; Chen and Guo, 1997; Garver and Guo, 2000; Xiao, *et al*., 2005; Lee and Guo, 2006). For phage λ, it has been found that the target component of gpA binding is the connector protein, because double mutation studies with the gpA and connector revealed that a connector mutation can suppress a gpA mutation that hinders gpA binding to procapsid (Yeo and Feiss, 1995). In bacteriophage T3, six molecules of gp19 bound to procapsids at a saturating amount of gp19, while the gp19 did not bind to procapsids lacking a connector (the gp8-defecient procapsid) (Fujisawa *et al*., 1991). In phi29 it was found that pRNA was cross-linked to connector, not to the capsid protein gp8 (Valle *et al*., 1999; Garver and Guo, 2000; Ibarra *et al*., 2000; Guasch *et al*., 2002; Hoeprich and Guo, 2002). N-terminal truncation studies on the connector of phi29 revealed that pRNA binds the N-terminus of the connector protein gp10 (Xiao, *et al*., 2005; Sun *et al*., 2006). Binding of phi29 gp16 to the packaging motor depends on the binding of pRNA, because the central domain of pRNA is for connector binding (Reid *et al*., 1994), while the 5′/3′ paired helical region is responsible for the recruitment of gp16 (Hoeprich and Guo, 2002; Lee and Guo, 2006).

### DNA packaging enzymes

All DNA packaging motors of dsDNA viruses involve one pair of non-structural components, classified into one of two categories according to their role in DNA packaging. The larger component containing the conserved ATP-binding motif is involved in procapsid binding, while the smaller component binds DNA (Guo *et al*., 1987a), as confirmed by subsequent studies in many viral systems. Procapsid binding components in well-studied phages include gpA in λ (Weisberg *et al*., 1979; Yeo and Feiss, 1995), gp12 in phi21 (Feiss *et al*., 1985), gp17 in T4 (Rao and Black, 1988), gp19 in T3/T7 (Yamagishi *et al*., 1985), g2p in SPP1 (Gual *et al*., 2000), g2p in SF6 (Chai *et al*., 1992), p9 of PRD1 (Stromsten *et al*., 2005), gp2 in P22 (Casjens *et al*., 1992) and gpP in P2/P4 (Rishovd *et al*., 1998). The DNA interacting components include gpNu1 in λ, gp1 in phi21, gp16 in T4, gp18 in T3/T7, g1p in SPP1, g1p in SF6 and gp3 in P22. The DNA packaging enzymes of eukaryotic viruses, such as herpes virus and adenovirus, are very similar to that of bacteriophages. Cytomegalovirus contains a pair of DNA packaging proteins pUL56 and pUL89 (Thoma *et al*., 2006). HSV also uses a pair of terminases, pUL15 and pUL28 (Koslowski *et al*., 1999; White *et al*., 2003; Yang and Baines, 2006). They assemble into the holoenzyme to work as the DNA packaging enzyme. The pair of DNA packaging proteins for adenovirus is IVa2 and the L1 52/55 kDa proteins (Zhang and Imperiale, 2003; Ostapchuk *et al*., 2005). However, there is still controversy in the adenovirus system concerning whether the DNA is packaged into preformed capsids. Although adenovirus has been thought to package its genome in a similar way as bacteriophages (Ostapchuk and Hearing, 2005), some evidence has been provided showing that the adenovirus genome is condensed with histone-like viral proteins (core protein pVII) prior to being packaged followed by the capsids assembly around the condensed viral genome (Lischwe and Sung, 1977; Zhang and Imperiale, 2003).

In phi29 related phages, three non-capsid components were involved in DNA packaging: gp3 that binds to the 5′-end of the DNA, the DNA-packaging protein gp16 and six pRNAs. Both pRNA and gp16 bind ATP independently with different affinity. As noted above, recent studies reveal that the central domain of pRNA binds to the 14 amino acids at the terminal of connector proteins gp10 (Xiao, *et al*., 2005) and gp16 binds to the 5′/3′ proximate domain of pRNA (Lee and Guo, 2006) (Fig. 1C). Therefore, despite the fact that pRNA is not a protein, as other terminases are concerned, pRNA itself, or the pRNA/gp16 complex, can be regarded as the counterpart of a large subunit of terminases, because it interacts with both the connector of the procapsid as well as ATP (Shu and Guo, 2003a; Xiao, *et al*., 2005). Gp16 interacts with DNA depending upon DNA structure other than the specific sequence in the dsDNA substrate (Lee and Guo, 2006). This finding is important because it implies that gp16 is not only involved in DNA contact in the initiation step, but is also a processive factor that keeps contacting the DNA during the entire DNA translocation process (Shu and Guo, 2003b).

## Stoichiometry of the components of the packaging motor

DNA or RNA packaging involves relative motion at the interface between the nucleic acid and motor components. An analogy can be deduced from a group of nucleic acid-binding proteins that play a similar role in DNA or RNA riding, translocating, sliding or tracking related to DNA or RNA replication, translocation, recombination and repair as well as RNA transcription initiation, extension and termination. Models of this group include *E. coli* DNA polymerase III holoenzyme, helicase (West, 1996; Niedenzu *et al*., 2001), the transcription terminator Rho (Burgess and Richardson, 2001), E1 replication initiator of bovine papillomavirus (Sedman and Stenlund, 1998), DNA polymerase processivity factors, thioredoxin of *E. coli*, sliding clamp/clamp loader complex PCNA in humans (Krishna *et al*., 1994; Gulbis *et al*., 1996; Guo *et al*., 1998; Ellison and Stillman, 2001) or the β subunit in *E. coli*, and gp45 in bacteriophage T4. Most members of this group are hexamers that encircle the DNA or RNA (Herendeen *et al*., 1992; Geiselmann *et al*., 1993; West, 1996). Their interaction with DNA or RNA could, in some cases, trigger ATP hydrolysis to release energy for conformal transitions. These components can be categorized into two subsets, one that only binds to nucleic acids and the other that acts catalytically on nucleic acids. The common feature of these two subsets is that they interact with DNA or RNA in a polymeric ring-shaped morphology.

When evidence of the pRNA hexamer was uncovered, the author instantly proposed that viral DNA packaging might have something in common with DNA replication and RNA transcription, and speculated that the mechanism of viral DNA packaging might be enlightened by the mechanism of DNA or RNA replication (Guo *et al*., 1998). This speculation caused a number of significant supportive and sympathetic responses (de Haas *et al*., 1999; Guasch *et al*., 2002). Sliding clamps are placed on DNA by clamp loader complexes helping polymerases to overcome the problem of torque generated during the extension of double-stranded helical DNA. ATP is required for its binding to dsDNA and for opening the clamp. The crystal structure reveals a spiral structure in the clamp loader with a striking correspondence to the grooves of the helix of dsDNA, which suggests a simple explanation for how the loader/DNA helix interaction triggers ATP hydrolysis and how it releases from the sliding clamp (Oyama *et al*., 2001). The mechanism might provide hints for understanding the role of the DNA packaging enzyme and its connector.

Additionally, it has been suggested that DNA packaging enzymes, including gp16 of phi29, might be one of the members in the AAA+ family of proteins, which contain universally conserved ATP binding domains and a high level of processivity (Fig. 1C) (Chemla *et al*., 2005; Lee and Guo, 2006). The AAA+ family members share a highly conserved ATP binding domain (Walker A motif), contain ATPase activity, and form a hexamer or pentamer in the presence of Mg^2+^ ions (Niedenzu *et al*., 2001; Li *et al*., 2003). Many of them also have been found to interact with DNA. The NTP hydrolysis activity of helicase is from 10 to 100 times lower in the absence of DNA than in its presence. This allosteric effect is similar to that of viral DNA packaging enzymes (see below).

**Fig. 2:**
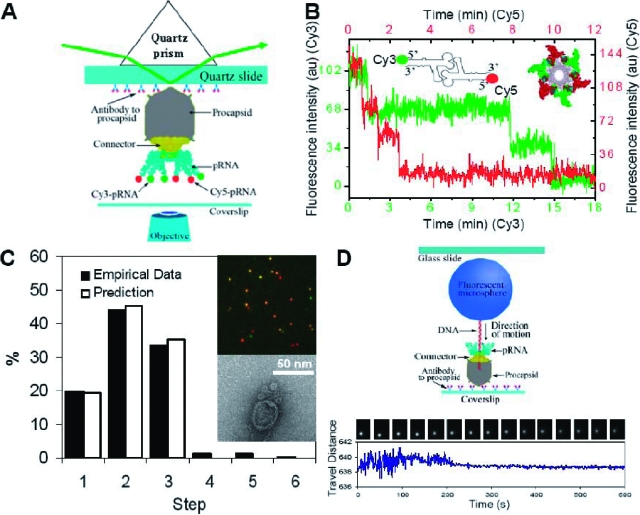
Single molecule studies on bacteriophage phi29 DNA packaging motor (adapted from (Shu *et al*., 2007) with permission from the European Molecular Biology Organization). A–C. Single molecule dual-view imaging for pRNA counting on the motor. (A) Experimental design. (B) Single molecule counting of Cy3 (green) or Cy5 (red)-labelled pRNA via step photobleaching. Each step represents the presence of single Cy3 or Cy5 on pRNA. (C) Single molecule dual imaging of procapsid containing Cy3-pRNA (green spots), Cy5-pRNA (red spots) or both Cy3 and Cy5-pRNA (yellow spots). The histograms of procapsid with Cy3 photobleaching steps were compared with the theoretical histogram predicted to have three Cy3 pRNA with a labelling efficiency of 70%. The stoichiometry of ferritin-conjugated pRNA on procapsid also revealed by EM. D. Direct observation of phi29 DNA translocation with gradual reduction of swinging range (distance) of the fluorescence microsphere at real time. The experimental design and the frames of the image are also shown.

The aforementioned common feature addresses questions related to the stoichiometry and symmetry of the components in viral DNA packaging motors. The connector is a dodecamer that bears a sixfold symmetry. Phi29 pRNA forms a hexameric ring encircling the DNA (Chen and Guo, 1997; Guo *et al*., 1998; Zhang *et al*., 1998; Guasch *et al*., 2002; Shu *et al*., 2007) (Fig. 2). Phage T3 terminase is a complex consisting of gp19 and gp18, and six copies of gp19 bind to the procapsid (Morita *et al*., 1995). In λ, one gpA and two gpNu1 form a heterotrimeric complex as a prototype, of which four protomers further assemble into an oligomeric ring-shaped motor (Maluf *et al*., 2006). In phage T4, gp16 was found to form stable octameric rings with an approximate diameter of 8 nm and a central channel of approximately 2 nm. The gp16 of T4 preferentially binds dsDNA containing gene 16 coding regions and stimulates the formation of higher-order oligomers of the large terminase subunit (gp17). The stoichiometry of phage SPP1 terminase has been proposed to be two g1p to one g2p that are present in the motor complex. In HCMV, the large subunit pUL56 has been observed as a U-shaped open ring dimer under EM (Savva *et al*., 2004). As suggested, the dimerized open ring might be able to bind DNA, then close subsequently. It is a role similar to PCNA, a DNA loader, as discussed above. In addition, the 20 amino acid sequences in the small subunit pUL89 are critical for the interaction with the large subunit pUL56 (Thoma *et al*., 2006).

In phi29, there has been a long-standing fervent debate regarding whether the pRNA ring is composed of five or six copies, which is important because different observations led different speculations of packaging models (see section 5). As the connector and the capsid shell holds six- and fivefold symmetry, respectively, the stoichiometry of five or six could lead to different explanations of motor mechanisms regarding which is the foothold for pRNA and which is the rotor and which is the stator, if the motor rotates. The debate has been settled by the application of a newly designed single molecule dual colour imaging system (Fig. 2) (Shu *et al*., 2007). Single molecule counting clearly identified the quantized photobleaching steps from the single fluorophore-labelled pRNA in procapsids, which led to a conclusion that the phi29 motor contains six copies of pRNA before and during DNA translocation (see section 6). In addition, EM imaging revealing six ferritins bound to the pRNA ring supports this conclusion. Once six pRNAs bind to the procapsid, they stay on the motor to carry out the DNA translocation task. This finding agrees with other studies reported earlier. In 1998, two labs (Guo *et al*., 1998; Zhang *et al*., 1998) demonstrated independently that pRNA forms hexamers as part of the phi29 motor. Repeated biochemical studies revealed the copy number of pRNA is the common multiple of 2 and 3, which is 6 (Guo *et al*., 1998). Mixing six different inactive pRNA monomers engineered to form a hexameric ring generates a high level of virus assembly activity (Guo *et al*., 1998; Zhang *et al*., 1998). It was found that dimers are the binding unit and the building blocks of hexamers, and thus it is not easy to account for how a pentamer could be formed by an even number of binding units (pRNA dimer) (Chen *et al*., 2000) on a targeted base with even-numbered symmetry (the dodecameric connector). It was also found that purified pRNA trimers have the highest specific activity comparing to the monomers (Shu *et al*., 2007). Carrascosa and coworkers also reported a finding of pRNA hexamer by cryo-EM (Ibarra *et al*., 2000). One argument for the five-pRNA speculation is that pRNA hexamers are formed initially, but after binding, one of the pRNAs dissociates from the procapsids, leaving five pRNAs still bound (Simpson *et al*., 2000). However, recent studies found that six pRNA remains on the motor that is actively translocating DNA, as found in the single molecule dual view of isolated motors in an intermediate stage containing pRNA labelled with green (Cy3) and DNA labelled with red (Cy5) (Shu *et al*., 2007). The one pRNA departure hypothesis was argued against by the finding that covalently linked pRNA dimers are active in DNA packaging (Chen *et al*., 2000). The hexamer view point was further supported by the finding that pRNA binds to the N-terminus of the connector protein gp10. As noted earlier, UV cross-linking of pRNA/procapsid or pRNA/connector complex proved that pRNA was specifically bound to the 12-fold connector, but not to the fivefold capsid protein gp8 (Garver and Guo, 2000).

## DNA substrate serving as the bolt or axle of the motor

One important consideration on the motor mechanism is whether DNA is geared in, serving as a bolt for a nut, or pushed or pulled in. DNA is involved in packaging in two ways: (i) the recognition of a specific sequence of DNA by the motor components for the initiation of DNA packaging and (ii) the successive interaction with the motor component during DNA translocation. Understanding the DNA structure is critical for DNA translocation and will help answer the questions regarding the action mechanism of motors, including DNA supercoiling, gyrase activity, or the contact between the wall of the motor channel and the DNA axle. Single chain breaks occur with variable frequencies at fixed sites of the genome of phage T5 and packaged T7 DNA (Khan *et al*., 1995). *In vitro* studies showed that gaps and single-stranded DNA stall or block the packaging, while nicks (breaks) can be tolerated in T3 (Fujisawa *et al*., 1987) and phi29 (Moll and Guo, 2005). However, nicks were reported to cause a reversible blocking to the completion of T4 packaging in ligase-deficient mutants. These stalled procapsids can be rescued by restoring ligase. Other mutations in early T4 genes, including the gene for topoisomerase II subunit gp39, also cause aberrant DNA structures that cannot be translocated. In the absence of T4 gene 49 endonuclease VII, long heteroduplex loops up to 150 bp can be tolerated and packaged. UV-damaged T4 DNA can be packaged, while UV-induced damage shows reduced packaging efficiency *in vivo*. In λ, DNA from cI^+^ phage was annealed with DNA from phages carrying small insertion or deletion mutations in the cI gene. Heterologies of 19 bp but not 26 bp can be packaged efficiently (Pearson and Fox, 1988). Subgenomic fragments with overlapping sequences of the adenovirus genome can also be packaged. However, some less characterized bacteriophages seem to possess considerable tolerance for structural abnormalities in the DNA they package. Mycobacteriophage I3, for example, was found to contain several single-stranded gaps in its packaged dsDNA genome (Reddy and Gopinathan, 1986), and *Rhodopseudomonas sphaeroides* bacteriophage RSI DNA is heterogeneous in length and contains nicks and gaps (Donohue *et al*., 1985). Extended gaps were also found in DNA packaged by Erwinia herbicola phage ErhI (Kozloff *et al*., 1981). Overall, the packaging machine might tolerate certain alterations and deviations of DNA substrates, but certain possible coupled or non-coupled DNA repair mechanisms *in vivo* make it difficult to study the structural requirement of DNA packaging *in vivo*.

How can the viral motor only package its own genomic DNA, but not non-specific DNA? Most viral genomic DNA or RNA contains a specific sequence as a signal for recognition. The junction area of λ concatemeric DNA contains three functional regions, known as cosN (a nicking sequence), cosB (a binding sequence) and a cosQ, required both to initiate and to terminate packaging (Catalano *et al*., 1995). The three cosB subsites, R1, R2 and R3, are the specific binding targets of gpNu1, while gpA interacts with the cosN region. IHF binds I1 elements located between the R1 and R2 elements within cosB, which causes an almost 180° bend in the duplex DNA (Fig. 1B) (Ortega and Catalano, 2006). This is critical to the introduction of gpA into the gpNu1 dimer to assemble it into an active holoenzyme. Once the holoenzyme is formed, gpA binds to cosN to nick and separate the DNA duplexes (de Beer *et al*., 2002). gpA introduces two symmetric nicks at the cosN region to generate two 12-nucleotide 5′ overhang ends. After separation from the mature right end, the terminase remains associated with the mature left end to form a complex I that binds a procapsid before DNA translocation. It is through allosteric interactions that ATP stabilizes binding of gpNu1-DBD to DNA, while binding of DNA stimulates ATP hydrolysis activity of gpNu1 (de Beer *et al*., 2002). Once their holoenzyme turns into an active packaging motor, gpA binds DNA non-specifically, leading to processive packaging. In SPP1, the *in vitro* packaging of DNA is independent of the pac signal, suggesting that the formation of free DNA ends by pac cleavage *in vivo* is the rate-limiting step in the processive headful packaging (Oliveira *et al*., 2005). In adenoviruses, IVa2 and L1 52/55 kDa protein recognize the packaging sequence, ‘A’ repeats, together with a host component, such as CCAAT displacement protein (Ostapchuk *et al*., 2005). Two terminase subunits of HSV, pUL15 and pUL28, also recognize the specific sequence on the DNA and the dodecameric portal pUL6 to translocate DNA followed by the cleavage of one unit of genome (White *et al*., 2003).

The direction of DNA packaging generally starts at the end opposite the one first ejected, usually referred to as left end to right end (the end ejected first) of the genome. Packaging of concatemeric DNA is directional and processive for both terminase cutting and headful cutting. The adenoviral genome is also packaged from the left end; however, non-specific DNA is not absolutely excluded although such a signal is preferably selected. In the absence of its own genomic DNA, non-specific DNA could be packaged with decreased efficiency.

Regardless of the size of the viral genome, DNA packaged inside the capsid is condensed to near liquid crystalline density, concentrated 30–100-fold to 500 mg ml^−1^ (Hohn, 1976). Cryo-EM and X-ray diffraction showed the structure of DNA packaged within the capsid in T7, P22 and T4, where encapsulated DNA was organized as a spool in concentric rings (Cerritelli *et al*., 1997; Lander *et al*., 2006; Chang *et al*., 2006). However, aside from phi29, few investigations have reported on the conformational requirements of DNA substrates that can be packaged. Natural DNA in solution is generally present in the supercoiled or relaxed form, which suggests that the helical nature of the B-form is the preferred conformation. Some viruses, including adenovirus and phi29, use one set of the genome as the packaging substrate. Their genomes contain a terminal protein covalently attached to the 5′-end. However, many dsDNA viruses produce concatemeric DNA during replication. These viruses use one of two mechanisms to terminate the packaging. Some viruses, such as T4, P22 and P1, use the ‘headful mechanism’, while others recognize the specific sequences as the termination signal. A ‘headful mechanism’ is defined as the stuffing of DNA into a protein shell until it is filled; thus, the capacity of the viral procapsid can be determined by the size of the packaged DNA. A recent study on P22 from the asymmetric reconstruction of package capsid by cryo-EM proposed that tightly spooled DNA causes a conformational change around the portal, which might act as a trigger for packaging termination (Lander *et al*., 2006). Phages λ, P2, P4, T3 and T7 recognize special sequences as the packaging termination signal. In λ, the translocating terminase complex nicks the DNA duplex at cosN when encountering the next downstream cos site, and then separates the duplex strands to terminate the genome packaging.

## Energy source and driving force of DNA packaging motor

In spite of the differences among motors in geometry and stoichiometry, as well as their function or role in nature, there are similarities in terms of energy conversion from chemical into mechanical form via repetitive conformational changes of motor components. Some biomotors are powered by hydrolysing an energy-rich bond of ATP, as combustion engines break down the molecules in petroleum distillates. Other biomotors are driven by the flow of protons along a proton gradient, i.e. an electrochemical potential across a membrane, just as electric motors are powered by the flow of electrons along an applied voltage. Both types of energy conversion have long been inferred to the study of viral genome-packaging motors.

Viral packaging motors use chemical energy derived from the hydrolysis of ATP. The active sites on motor proteins bind ATP and catalyse its decomposition to ADP and inorganic phosphate (P_i_), thereby releasing a significant quantity of energy and inducing conformational changes in the motor structure that ultimately results in work performed by the motor. This catalytic process is then restarted by the next ATP molecule. By doing so, the motor protein can continue further movement. It is ATP hydrolysis that provides the driving force to viral DNA packaging motors in order to overcome such an entropically unfavourable DNA packaging reaction. The ATP-binding consensus sequences of the DNA-packaging enzymes of many viruses were summed up as: Basic aa Hydrophobic region GxxGxGKS (or T) xxxxxxx Hydrophobic aa (Guo *et al*., 1987b). Mutation of a single amino acid at this region could completely abort ATP binding and hydrolysis, thereby completely inhibiting the DNA packaging. λ gpNu1 and phi29 pRNA itself does not show ATPase activity, rather it shows DNA-dependent low affinity ATPase activity only in λ holoenzyme or pRNA/gp16 complex. However, gpA of λ and gp16 of phi29 show a high-affinity ATPase activity (Yang *et al*., 1999) (T. L. Lee and P. Guo, unpubl. data). The ATP binding to gpA is essential to allow a proper positioning of terminases on the cos cleavage site. While ATP hydrolysis is not required for the endonuclease activity of gpA to nick on cosN, its helicase activity that separates the nicked duplex needs ATP hydrolysis. Interestingly, it was proposed that the N-terminal region in gpA might possess additional non-Walker motifs for the ATPase activity that is responsible for actual DNA translocation, because a change in the putative Walker A motif only affects helicase activity, not overall packaging activity (Hang *et al*., 2000). In SPP1, at the different g1p/g2p stoichiometry, the terminase shows different ATPase and endonuclease activity. When g2p interacts with g1p in a manner of g1p_2−3_g2p_1_, its ATPase activity is enhanced (Camacho *et al*., 2003). Two separate ATPase activities existed in T3. One of these, called pac-ATPase, required viral DNA, while the other, termed non-pac ATPase, was stimulated by non-packagable DNA (i.e. single-stranded or circular) or RNA (non-specific). However, the non-pac ATPase activity continued, after initial activation, in the absence of viral DNA. These individual ATPase activities can be stimulated by other components (Morita *et al*., 1993). In phi29, the ATPase activity of gp16 can be stimulated either by pRNA or DNA. Similarly, procapsids with pRNA stimulate the ATPase activity of gp16 10-fold, as compared with the lack of stimulation by procapsids alone. The ATPase activity was maximally stimulated only by all packaging motor components including pRNA, procapsid, gp16 and DNA-gp3 (Guo *et al*., 1987b; Shu and Guo, 2003a). Recent single molecule studies revealed that the phi29 packaging motor has an initial rate of 100 bp s^−1^ with an external load of 2.2 μm polystyrene microspheres on the DNA, and that the power corresponds to a force of 57 pN (Smith *et al*., 2001), which is one the most powerful bionanomotors ever constructed to date. Quantification of ATP consumption in DNA packaging in the defined *in vitro* DNA packaging system of phi29 shows that every ATP hydrolysis corresponds to 2 bp of movement of DNA (Guo *et al*., 1987b). Similarly, in bacteriophage T3, one ATP molecule was required to package 1.8 bp of T3 DNA (Morita *et al*., 1993). It was also found in phi29 that both the DNA packaging protein gp16 and pRNA possess a higher binding affinity for ATP than for ADP or AMP (Shu and Guo, 2003a). As in dsDNA bacteriophages, HCMV pUL89 does not show ATPase activity by itself, but it enhances the ATPase activity of pUL56 via holoenzyme formation (Hwang and Bogner, 2002). It has also been suggested that the A32 gene might be a putative terminase, because it possesses the conserved ATP binding motif similar to IVa2 of adenoviruses (Koonin *et al*., 1993). These observations indicate that the binding of ATP is central to cause conformational changes of the motor to transfer chemical energy (ATP) into motion (packaging). Single molecule studies implied that the conformational change of the motor component might be correlated with the release of inorganic phosphate to generate the power stroke on the DNA, rather than the ATP binding step (Smith *et al*., 2001).

## Models for the mechanism of a viral DNA packaging motor

A number of models have been proposed to describe the mechanism of DNA translocation.

### Gyrase-driven packaging model

Based on the fact that a T4 DNA ligase mutant ruins DNA packaging activity, it was hypothesized that T4 terminase gp17 introduces supercoiling to compress the translocated DNA near the apex of the portal, using ATP as an energy source for DNA gyrase. After entry, the supercoiling is relaxed by introducing nicks along the DNA. The model is supported by the finding that the packaged DNA in T7 and T5 contains nicks (Khan *et al*., 1995), and favoured by the report that phi29 gp16 introduced supercoiling after gp16 binds to DNA (Grimes and Anderson, 1997). However, it is not supported by the finding that nicked DNA of T7, T5, T3 and phi29 can be used as a substrate for packaging *in vitro* (Khan *et al*., 1995; Moll and Guo, 2005).

### Osmotic model and ratchet model

The osmotic model proposed that the difference between internal low pressure and outer high pressure generates osmotic pressure and serves as a driving force for DNA translocation (Serwer, 2003). The expansion of the procapsid lowers the osmotic pressure inside the capsid, and the connector acts as a ratchet to grab the packaged DNA. The model suggests that procapsids contract to expel the scaffolding protein and non-DNA molecules through the connector hole in order to maintain low internal osmotic pressure as packaged DNA increases. At the same time, the entry hole of the capsid narrows to trap the DNA to prevent losing the packaged DNA. It is proposed that energy from ATP hydrolysis is used to contract the capsid and pump out the non-DNA molecules. The later ratchet model, modified from the osmotic pump model, speculated that the presence of an electro-dipole central channel involving electrostatic motion complements the thermal ratchet mechanism using osmotic pressure-assisted oscillating thermal motion (Serwer, 2003). The force of DNA movement is proposed to proceed by Brownian motion (Astumian, 1997). The motor works as a ratchet that rectifies oscillating forces simply to ensure that DNA only moves in one direction. However, the model does not explain how the decreased osmotic pressure can drive DNA translocation when the procapsid is almost full and the internal pressure has increased. Furthermore, the model does not consider the finding that low ATP concentration correlates with a lower DNA packaging rate (Smith *et al*., 2001) and that the ATP binding consensus sequence is not found in the connector sequence, but in packaging enzymes with DNA-dependent ATPase activity (Guo *et al*., 1987b). Moreover, procapsid expansion has been reported in T4 (Jardine and Coombs, 1998), λ, P22, T7 and T3, while it is not necessary in phi29 (Bjornsti *et al*., 1983).

### Connector rotating thread model

The symmetry mismatch between the portal vertex of procapsid (fivefold) and the connector (sixfold) was proposed to enable the rotation of the connector, producing a driving force to translocate the DNA through the connector channel (Hendrix, 1978). Weak interaction between the procapsid and connector due to their symmetry mismatch decreases the potential energy barrier to rotate the connector along the procapsid. The translocation of DNA through the axial hole of the portal vertex is much like a threaded rod moving through a nut, and thus DNA packaging could be achieved by using the ‘threaded’ helical nature of dsDNA by an ATP-driven spinning connector. It requires the perfect fit between the inside surface of the connector channel and the groove of DNA as well as a certain restraint for holding the end of DNA inside the procapsid so as not to rotate along with the rotating connector. However, there is no obvious evidence to validate this intriguing and popular model that was proposed three decades ago. Futhermore, the high affinity between connector and pRNA would not support rotation at the junction of these components (Robinson *et al*., 2006).

### Supercoiled DNA wrapping model

This model proposed that supercoiled DNA wraps around the portal vertex and the rotation of the connector allows DNA to pass into the procapsid. It is based on the finding that the phi29 connector preferentially binds supercoiled rather than linear DNA (Grimes and Anderson, 1997). Topoisomerase I treatment removes supercoils and nicks, revealing supercoil numbers equivalent to the bound connector (Turnquist *et al*., 1992). The model is unique in that DNA wraps around the outside of the connector and that DNA enters the procapsid via the outside of the connector rather than the central channel. However, this model is not favoured by recent structural data showing that DNA is likely positioned in the central channel of the connector when partially packaged procapsids were analysed by cryo-EM (Simpson *et al*., 2000).

### Sequential action model

This model is based on the function of six pRNA of phi29. It proposed that the relative motion of the hexameric pRNA ring against the pentameric vertex of the capsid shell could provide a driving force for DNA translocation (Chen and Guo, 1997). Analogous to a six-cylinder car engine, the sequential firing of six pRNAs is a possible way to rotate the motor. The pRNA contains two domains, the central domain binding to the connector (Xiao, *et al*., 2005) and the 5′/3′ proximate domain serving as a moving arm interact with gp16 (Lee and Guo, 2006). The gp16/pRNA complex serves as an ATPase and possesses at least two conformations. Alternating between contraction and relaxation, each member of the hexameric complex driven by ATP hydrolysis helps generate torque to rotate the DNA translocation machine, with one ATP consumed for 2 bp of DNA translocation per step (Guo *et al*., 1987b). DNA serves as a bolt, and the driving of the motor for DNA translocation involves the double helix of DNA. The connector/pRNA complex resembles hexameric PCNA (proliferating-cell nuclear antigen), and gp16 is an AAA^+^ type protein that interacts with DNA (Fig. 1C).

The sequential model explains why the pRNA is so sensitive to mutation; a pRNA mutation will be amplified by six orders of magnitude after six consecutive steps, resulting in the complete loss of DNA packaging activity despite a small alteration. Replacing any one of the six pRNAs in the sequence with an inactive variant completely blocked the motor function, strongly supporting the speculation that each subunit takes turns mediating successive steps of packaging. Computation of the probability of combination between wild type and mutant pRNA and experimental data of competitive inhibition favours the sequential action model. This model was supported by the finding that ATP induces conformational change in pRNA, and that pRNA loop/loop interaction is essential to pass the signal from one pRNA to the other. Although, so far, there is no direct evidence arguing against the model, the direct observation of sequential action of hexameric pRNAs has not yet been demonstrated.

### Electro-dipole central channel model

The connector rotation hypothesis based on the fivefold/sixfold symmetry mismatch (Hendrix, 1978) can be linked to the inner shape of the central channel of the connector, as proposed (Guasch *et al*., 2002). The 3D crystal structure of the phi29 connector showed that the inside surface of the central channel is mostly electronegative, which does not favour close contact to the electronegative phosphate backbone of DNA, and that the average diameter of the channel (35 Å) is wide enough to give a loose sliding displacement to DNA (23 Å). Two lysine residues 20 Å apart along the central channel were found to form two lysine rings inside the channel (Guasch *et al*., 2002). It is likely that two phosphates on the adjacent major grooves of DNA contact each of the lysine residues during DNA translocation. The model suggests that a 6° rotation of the connector displaces the lysine–phosphate interaction to the next step, corresponding to 1 bp movement of DNA. The consecutive rotation of the connector is required so that lysine-phosphate pairs are re-established 1 bp at a time, consistent with the fivefold/sixfold symmetry mismatch hypothesis (Hendrix, 1978) and the experimental data showing that one molecule of ATP is required to package 2 bp of DNA (Guo *et al*., 1987b). The model does not explain whether the ATP-generated force acts on the connector to drive DNA translocation, or on the DNA to make the connector rotate in a passive way. In addition, the model suggests that connector subunits are not rearranged during DNA displacement.

### Connector contraction hypothesis

This model was proposed for T3 (Morita *et al*., 1995) and subsequently for phi29 (Simpson *et al*., 2000). A 12° rotation of the narrow end of the connector leads to lengthwise expansion of the connector via a slight change in the angle of the long helices, and the wide end of the connector follows the narrow end. Such a ‘following’ allows structural relaxation and contraction during DNA translocation. It was proposed that the power stroke comes from the pRNA, which is the stator that uses the procapsid as its standing (footing) base. The symmetry mismatch between pRNA (fivefold) and connector (sixfold) gives rise to discrete anticlockwise (view down the connector axis towards the procapsid) 12° rotational steps of the connector. One complete turn of the connector will transfer 60 bp of DNA into the procapsid. However, this model is contrary to the finding that pRNA uses N-terminus of the connector but not the capsid protein as its foothold (Xiao *et al.*, 2005). In bacteriophage T3, the large subunit of terminase, gp19, did not bind to procapsids lacking a connector (Morita *et al*., 1995), which indicates that most packaging motor components contact the connector, not the portal vertex around the connector. In addition, this model is based on the observation by Simpson *et al*. (2000), in that the pRNA had been observed as the fivefold symmetry to explain that the vertex of the capsid protein and the pRNA was the stator. Recent single molecular studies have confirmed that the pRNA ring is a hexamer (Shu *et al*., 2007).

The other possible model relevant to the fixed connector hypothesis could involve the pushing or injection of the DNA by the DNA packaging protein. In this model the connector is a stator, while the DNA packaging protein itself is responsible for the translocation. The packaging proteins insert a certain length of DNA, and then shift to bind to a far distal region of the DNA and insert an additional region.

All the aforementioned DNA packaging models are very intriguing, but none of them has been supported by conclusive experimental data. A recent report (Baumann *et al*., 2006) that fusion of the connector protein with C-terminal GFP (located inside the capsid) and with N-terminal capsid binding HOC protein (located outside the capsid) did not affect T4 DNA packaging challenges the validity of the portal rotation theory. As pointed out by the authors, however, it is uncertain whether the packaging machine generates a power stroke sufficient to overcome the binding stability of HOC-tethered docking between the connector and the shell, because it is a non-covalent interaction (Maluf and Feiss, 2006). In fact single molecule packaging analysis may be necessary definitively to demonstrate or exclude portal or other (e.g. terminase-based) rotational packaging motors. Nevertheless, for any virus, even if the motor is rotating, it is currently still unclear about which component is the rotor and which one is the stator.

## Single molecule studies on DNA packaging motors

Recent advances in single molecule fluorescence microscopy have provided a new way to understand motor mechanisms by directly counting motor components and observing motion events (Fig. 2) (Shu *et al*., 2007). It allows the analysis of individual motor components, as opposed to the ensemble averaging of the measurements from a massive population of homogenous molecules that are motionless or requiring synchronous motion (Vale *et al*., 1996; Ishijima *et al*., 1998; Ha, 2001; Rueda *et al*., 2004). Single molecule approaches also allow the direct observation of physical behaviours to answer many questions as noted earlier, including how the chemical energy is converted into the physical motion and the efficiency of energy conversion (Adachi *et al*., 2000; Hirono-Hara *et al*., 2001; Yasuda *et al*., 2001), how the force is generated through the gearing of motor structures (Svoboda and Block, 1994), how individual motor components are involved in and respond to chemical reactions in real time (Zhuang *et al*., 2000; Ha *et al*., 2002; Shu *et al*., 2007), how the motion starts and continues; how each motor component responds to the applied force (Chemla *et al*., 2005; Smith *et al*., 2001) and how the conformational change of each motor component contributes to force generation (Chemla *et al*., 2005; Jankowsky, 2005; Myong *et al*., 2005; Rondelez *et al*., 2005; Seidel *et al*., 2005). Unveiling the clues to these questions will make it possible to elucidate the properties of bionanomotors, and will also help to design novel nanodevices (Soong *et al*., 2000; Tomishige *et al*., 2002; Bringmann *et al*., 2004; Pomerantz *et al*., 2005; Fletcher *et al*., 2005). The following approaches have been successfully applied to the study of DNA packaging motors.

### The force measurement of DNA packaging motor using optical tweezers

Optical tweezers, or laser traps, have been used to catch or trap a small particle by the force generated from laser radiation pressure (Nambiar *et al*., 2004). The force on the object in the trap can then be determined. It was used to study the phi29 DNA packaging motor. A microsphere was connected to the distal end of phi29 DNA labelled with a biotin, and the procapsid that used to package the DNA was immobilized with antiphi29 antibody (Smith *et al*., 2001). It was found that the motor can package DNA against an internal force of 57 pN, implying that the phi29 motor is the most powerful motor ever constructed to date. The result also indicates that the viral capsid containing the tightly packed DNA should be able to withstand the substantial internal pressure. It has been suggested that the tensile strength of the phi29 procapsid might be equivalent to an aluminium alloy. The speed in DNA translocation was found to be about 100 bp s^−1^. One interesting observation in this single molecule study is the finding of the sliding of DNA during translocation. Although the mechanism for such DNA sliding has not been elucidated, it implies that the length of the packaged DNA might not simplify a direct proportion of step size multiplying by steps. These optical tweezer studies were also used for direct kinetic characterization of the DNA packaging reaction with ATP (Chemla *et al*., 2005). It was shown that the force-generating step for the DNA translocation is not associated with ATP binding, but is possibly associated with phosphate release to generate ADP. In combination with previous bulk experiments (Trottier *et al*., 1996; Chen and Guo, 1997), the results indicate that multiple ATPase protomers are involved in the packaging reaction by sequential action, and each ATPase protomer might complete its ATP hydrolysis cycle before the next cycle start.

### Single molecule counting

As noted earlier, the stoichiometry determination is central to elucidate the mechanism of motor action. Due to the limitation of resolution power and sensitivity of light microscopy, direct counting of motor components has been difficult. Recently, a customized single-molecule dual viewing total internal reflection fluorescence imaging system with a top-prism (SMDV-TIRF) has been constructed (Shu *et al*., 2007). It includes a customized laser combiner, an optical fibre to deliver multiple laser beams, dual-view emission optics to split different wavelengths of emitted light, a cooling system to reduce thermodynamics and background noises, an electron multiplier detector, and a perfusion chamber with a prism on the top, instead of on the bottom to avoid to the leakage to the objective lens of the reflected laser light. This system produces stable signals with extremely low background for single fluorophore detection. Single fluorophore imaging enabled a clear identification of the quantized photobleaching steps in the phi29 pRNA labelled with a single fluorophore to conclude its stoichiometry within the motor. The stalled motors were restarted to observe the motion of DNA packaging in real time. Dual-colour detection in the stalled intermediates containing both Cy3-pRNA and Cy5-DNA, where red (Cy3)- and green (Cy5)-overlapped yellow-coloured complexes represent motors actively translocating DNA, showed that most of motor complexes contain six copies of pRNA before and during DNA translocation (Shu *et al*., 2007). It confirmed the previous biochemical data showing that the stoichiometry of pRNA is six.

### Direct observation of motor motion

Direct observation of motion in real time have been reported in other biomotors (Oda and Takanami, 1972; Svoboda and Block, 1994; Vale *et al*., 1996; Noji *et al*., 1997; Kato-Yamada *et al*., 1998; Ellis *et al*., 1999; Sambongi *et al*., 1999; Harada *et al*., 2001). Attaching a fluorescent microsphere at the end of phi29 genomic DNA has been employed to track the motion during DNA packaging (Shu *et al*., 2007). The attached fluorescent microsphere amplifies the signal to be directly observed by fluorescence microscopy. Its motion can be profiled in a 3-dimensional space (x, y and z axis). The DNA packaging intermediates were stalled by a non-hydrolysable γ-S-ATP, restarted by addition of ATP, and observed at real time by fluorescence microscopy. DNA migration caused the attached microsphere to show a gradual reduction in swing range. Finally, the motion stopped due to the physical restriction of DNA being completely packaged, and appeared under the CCD camera as a zero distance change from the reference origin.

## Conformational change, sequential action and asymmetric distribution of motor components

In considering motor action mechanisms, force production and component gearing are two key factors. Many macromolecules undergo a conformational change to generate force in executing tasks involving motion, rotation, or migration. In component gearing, sequential action is the effective way to ensure successive motion (Hendrix, 1978; Chen and Guo, 1997). Like the sequential action of a six-cylinder engine in a car, the sequential action of the pistons is the way to keep the motor rotating. If the six cylinders fired synchronously, the engine could not operate continuously.

Viral structure is typically viewed as symmetrical components, such as penton or hexon in the icosahedral architecture. Structural virologists have conventionally used the symmetrical averaging program for successful analysis of viral structures. However, such symmetrical averaging approaches might not be appropriate in the studies of the motion components in a DNA packaging motor. When the sequential conformational changes in the phi29 DNA packaging motor were reported, the possibility of asymmetric distribution among motor components was implied (Chen and Guo, 1997). If motor components act sequentially in a step-by-step process, with each component exerting its effect alternatively, the controlled contact between the adjacent motor components might be necessary to deliver the motion signal from one after the next adjacent one sequentially. In doing so, one component must move forward to initiate the next step of motion, like the movement of an inchworm. The question of whether a quasi-pentamer situation of pRNA hexamer exists is central to understand the motor mechanism, because it is related to whether it is part of the stator or the rotor. Conventional cryo-EM studies of virions have revealed impressive typical symmetric distribution patterns of viral structure. In the structural analysis of motor components in motion, however, it is obvious that the use of symmetry averaging for tailed bacteriophages only leads to the lack of detailed information, especially the portal and tail region and spooled DNA. When a certain symmetry is enforced to average all images, the final image would be reconstructed to the enforced symmetry. Thus, in phi29, it is possible that the quasi-pentamer image of pRNA hexamer is the result of asymmetric distribution of the hexameric pRNA promoted by conformation change and sequential action (Chen and Guo, 1997; Guo *et al*., 1998; Shu *et al*., 2007). Because the motor would be in motion at any given time, it might not be possible to freeze the motor component in a regular symmetrical polygonal distribution in position. The recent advances in asymmetric reconstruction studies for viral structures from cryo-EM would provide more detailed information about the asymmetric distribution of individual motor components (Chang *et al*., 2006; Jiang *et al*., 2006; Lander *et al*., 2006). In biological systems, force is generally produced by conformational changes of components involved. Recent asymmetric reconstruction approaches with the packaged P22 portal revealed that the tightly spooled DNA around the connector inside of capsid induces a significant conformational change in the connector (Lander *et al*., 2006). Such a physical change on the packaging motor was proposed to trigger a stop signal of DNA translocation in the headful-type viruses. In bacteriophage Epsilon 15, single particle electron cryomicroscopy without icosahedral averaging showed an asymmetric distribution of tail spikes with quasi sixfold symmetry (Jiang *et al*., 2006). Individual spikes were arranged with different orientations and heights, which causes a small tilt of tail structure over the fivefold portal vertex. It was suggested that the different extents to which individual spikes were bent reflected different conformational changes that could lead to the opening of the portal for DNA injection. In phi29, evidence supports the idea that the sequential action of pRNA is the result of a conformational change in each pRNA. The conformational change of pRNA during the transition from dimer to hexamer has also been predicted (Hoeprich and Guo, 2002). It also has been reported that the conformational change of pRNA is induced by Mg^++^, which is critical for dimer formation and ATP binding (Shu and Guo, 2003a).

## Packaging motors for viral genomic RNA

The dsRNA viruses, like bacteriophage phi6 and phi12, also package their genomic RNA into preformed procapsids using a packaging motor. The packaging motor of phi6 is a complex of a RNA-dependent RNA polymerase, P2, and a packaging motor component, P4, both of which reside at the fivefold portal vertex of the icosahedral packaging complex. The phi6 genome consists of three segments of dsRNA (segments S, M and L) located within a procapsid composed of four proteins (P1, P2, P4 and P7).

Plus strand segments are packaged first and then serve as templates for the synthesis of minus strands. Therefore, the packaging of minus strands only begins after the completion of the plus strand packaging. The whole packaging process is precise and serially dependent. For example, at the beginning of packaging, the procapsid forms binding sites on the outside for segment S; while S binds to the procapsid and is being packaged by the ATPase motor P4, the binding sites for S disappear and those for M appear. When M is completely packaged, the binding sites for M change to L. Therefore, the packaging of M is dependent on S, and the packaging of L is dependent on M and S. All three strands share a common packaging signal, pac, an identical 18-base sequence located at the 5′ ends of each RNA (Iba *et al*., 1982). Each segment also possesses a unique sequence 50 bases from the pac sequence. Various manipulations performed on the RNA fragments revealed that packaging ability is completely lost upon a minor change in the pac sequence, while an extensively deleted RNA molecule, containing an unmodified pac sequence, can still be packaged and function as a template for minus strands. However, plus strand segments with a mutation or truncation at the 3′ end block the synthesis of minus strands, even when packaging was not affected. Unusually long or short RNA can also be packaged. An RNA molecule of sequence S joined to M, or S joined to M and L, can be successfully inserted into the procapsids. A 1/5-size segment of the normal segment can be packaged in five copies (Onodera *et al*., 1995), which suggests that the total amount of RNA that can be packaged per segment is approximately equal, which is similar to the ‘head-full’ mechanism in dsDNA viruses. The pac sequence is analogous to that in SPP1 and other dsDNA viruses.

P4 forms hexameric multimers and possesses ssRNA-stimulated NTPase activity as well as helicase activity. The P4 hexameric motor translocates ssRNA through its central hole by sequential ATP hydrolysis in each subunit with the action of arginine fingers. Binding of ATP causes rearrangement of arginine fingers. Subsequently, Arg279 moves away from the binding pocket to form hydrogen bonds with Arg251. Arg279 is then released and inserted into the nucleotide binding site of an adjacent subunit and forms a hydrogen bond with the α-position phosphate of bound ADP. This stabilizes the next subunit to trigger hydrolysis of the bound ATP. The power stroke from ATP hydrolysis moves ssRNA-bound L2 loop down to translocate RNA. Moving of L2 loop downward breaks the Arg279/Arg251 interaction of the next subunit (Mancini *et al*., 2004). In this model, all of the subunits within the hexameric motor complex are capable of ATP binding and hydrolysis, unlike the hexameric F1-ATPase ring, where only a β subunit, not an α subunit, can hydrolyse the bound ATP.

## Viral assembly versus bottom-up approach in nanotechnology: applications of viral packaging motors in nanotechnology and nanomedicine

The novel and ingenious configuration of bionanomotors and other biological machines have inspired the development of biomimetics in nanotechnology. In nanotechnology, two approaches, the top-down and the bottom-up, have been used to create nanomaterials and nanodevices to fill the gap between the micron-scale materials, which are readily available, and molecules, which exist abundantly in nature. Virologists have carried out both the top-down and bottom-up approaches for centuries. Current understanding in viral assembly has reached the point where we can begin thinking about the possible applications of viral motors in nanotechnology and nanomedicine, as has been demonstrated of concepts in other biomotors (Soong *et al*., 2000; Hess and Vogel, 2001). Viral DNA packaging motors and their related components have potential applications, including the delivery of drugs or therapeutic DNA or RNA, tissue and organ repair, the diagnosis of diseases and the detection of pathogens (Khaled *et al*., 2005). It might be applied in other nanodevices such as nanoelectromechanical systems (NEMS) (Craighead, 2000), molecular sorters, intricate arrays such as chips for diagnostics or computations, molecular sensors, complex actuators and electronic or optical devices. The size of the viral DNA packaging motor is in the nanometer scale.

One viable option that has been pursued for the development of mechanical parts for nanotechnology is to incorporate the viral packaging motor or their constituent parts into nanodevices using conventional fabrication techniques.

Currently, many viral DNA packaging motors have been constructed (Guo *et al*., 1986; Rao and Black, 1988; Fujisawa *et al*., 1991; Hwang and Feiss, 1997; Oliveira *et al*., 2005). The added DNA can be packaged with very high efficiency into purified motor complex. Phi29 DNA-packaging motor has been confirmed as one of the strongest known nanomotors constructed *in vitro*, with a stalling force of 57 pN (Smith *et al*., 2001; Chemla *et al*., 2005). In addition, the use of ATP analogues, DNA oligos and magnesium chelating reagents or other regulatory aptamers to turn-on and turn-off the motor (Guo *et al*., 1987c; Smith *et al*., 2001; Shu and Guo, 2003b; Shu *et al*., 2007) make it possible to engineer a controllable motor in a nanodevice. Similar to other viral structural components, the building blocks of viral packaging motor also possess a unique intrinsic property to form oligomers with symmetrical orientation, which makes viral packaging motor components ideal candidates as building blocks in bottom up assembly (Bazinet and King, 1985; Carazo *et al*., 1986; Rishovd *et al*., 1998; Valpuesta *et al*., 2000; Shu *et al*., 2004; Agirrezabala *et al*., 2005; Guo, 2005; Khaled *et al*., 2005). For example, the pRNA of phi29 contains one pair of left and right interlocking loops. Strong tendency of loop/loop interaction results in the formation of dimers, trimers and hexamers. Using the principle of bottom-up assembly in nanotechnology and applying a palindrome sequence for self-linkage, phi29 pRNA has been used as a building block to construct nanomaterials with a variety of structures and shapes to form twins, tetramers, rods, triangles and arrays several microns in size. Such arrays are unusually stable and resistant to a wide range of temperatures, salt concentrations and pH (Shu *et al*., 2003; Shu *et al*., 2004). Phi29 pRNA property of controllable self-assembly into polymers has been used to construct polyvalent nanoparticles for the delivery of siRNA, ribozyme, drugs, fluorescent of radioactive markers or other therapeutic molecules to specific cells with potentials for the treatment of cancer, viral infection and genetic diseases. For example, one RNA subunit of the dimer, trimer or hexamer carries therapeutic RNA, such as a hammerhead or hairpin ribozyme, or antisense RNA, while the other RNA subunit carries ligands or aptamers that recognize specific cell surface receptors for an endocytotic delivery. Specific receptor-binding RNA molecules can be isolated through SELEX (Systematic Evolution of Ligands by Exponential Enrichment) (Ellington and Szostak, 1990; Tuerk and Gold, 1990). The use of such immunogenecity-free 20–40 nm particles holds promise for the repeated long-term treatment of chronic diseases (Guo *et al*., 2005; Khaled *et al*., 2005). The property of self-assembly into regular polygonal oligomers, for example, pentamers, hexamers, or dodecamers, has been and will be used for polyvalent detection. During early stages of cancer development, cancer cells dramatically express a variety of cell markers that required detection apparatus for multivalent detection (Guo *et al*., 2005).

One rising area in biology is the development of efficient, inexpensive and highly sensitive analytical tools that can be used to probe, analyse, interpret and manipulate single molecules, such as a single channel ion transportation pore or nanopore-based DNA sequencing device (Deamer and Akeson, 2000). It will recognize a single base pair, based on the electrical signals generated through the interaction of the bases of the DNA with a pore. The viral packaging motor has the potential to be developed into a DNA sequencing apparatus, because the DNA packaging process involves movement of the DNA through a few nanometer-scaled pore surrounded by packaging motor components that can be modified to accept chemical or electrical signals.

The application of viral packaging motors in nanotechnology and nanomedicine has been exemplified by the recent selection of the phi29 DNA packaging motor as an NIH national Nanomedicine Development Center, entitled Phi29 DNA Packaging Motor for Nanomedicine (see NIH website at http://nihroadmap.nih.gov/nanomedicine/fundedresearch.asp and NDC website at http://www.vet.purdue.edu/PeixuanGuo/NDC). The ultimate goal of the centre is to create biologically compatible membrane or polymer adapted motor chimeras embedded into liposome of cell membrane for passive or active delivery of DNA, RNA or drugs into specific cells.

## Concluding remarks and perspectives

Given the broad similarities found between different viruses, studies in genome encapsidation could provide clues for novel targets in antiviral therapy (Visalli and van, 2003; Trottier *et al*., 1996). Understanding the mechanism of viral DNA translocation might shine light on DNA translocation or RNA transportation through cell membranes, nucleic acid repair or proofreading, and DNA replication or RNA transcription. On the other hand, the current popular studies on the mechanisms of DNA or RNA translocation, the riding or tracking during RNA transcription, DNA replication or nucleic acid repair should be used as references in the study of viral DNA or RNA packaging (Guo *et al*., 1998; Lee and Guo, 2006). In addition, viral DNA packaging enzymes might be a part of the AAA+ family (Lee and Guo, 2006). Advancement in the studies on viral DNA packaging enzymes could be achieved by drawing lessons from the investigations on structure and function of the unique but widespread AAA+ family. The most feasible immediate approach to elucidate the structure and mechanism of a variety of viral motors is to determine the stoichiometry of motor components. Besides the conventional structural, chemical, genetic and biochemical approaches, it is essential to dissect the conformational changes and reveal images in real time. Growing advances in single molecule imaging and detection will provide powerful capabilities to determine the stoichiometry and motor mechanism. The phenomenon of coexistence of conformational change and sequential action has led to the new concept of the asymmetrical distribution of motor components (Hendrix, 1978; Chen and Guo, 1997). Because the DNA packaging motor is a motion machine and exciting data about an asymmetry have emerged most recently (Jiang *et al*., 2006; Lander *et al*., 2006), the courage to break the conventional symmetrical concept in structural virology could advance this field.
